# Choosing Surgery for Neurogenic TOS: The Roles of Physical Exam, Physical Therapy, and Imaging

**DOI:** 10.3390/diagnostics7020037

**Published:** 2017-06-23

**Authors:** David P. Kuwayama, Jason R. Lund, Charles O. Brantigan, Natalia O. Glebova

**Affiliations:** 1 Division of Vascular Surgery and Endovascular Therapy, Department of Surgery, University of Colorado Denver, Denver, CO 80045 USA; david.kuwayama@ucdenver.edu (D.P.K.); cbrantigan@drbrantigan.com (C.O.B.); 2 Ashbaugh Center for Physical Therapy, Denver, CO 80222, USA; jasonrlund@hotmail.com; 3 Section of Vascular Surgery and Endovascular Therapy, Department of Surgery, University of Colorado Anschutz Medical Campus, 12631 East 17th Ave, Room 5409, Mail Stop C 312, Aurora, CO 80045, USA

**Keywords:** neurogenic thoracic outlet syndrome, physical examination, physical therapy, imaging

## Abstract

Neurogenic thoracic outlet syndrome (nTOS) is characterized by arm and hand pain, paresthesias, and sometimes weakness resulting from compression of the brachial plexus within the thoracic outlet. While it is the most common subtype of TOS, nTOS can be difficult to diagnose. Furthermore, patient selection for surgical treatment can be challenging as symptoms may be vague and ambiguous, and diagnostic studies may be equivocal. Herein, we describe some approaches to aid in identifying patients who would be expected to benefit from surgical intervention for nTOS. We describe the role of physical examination, physical therapy, and imaging in the evaluation and diagnosis of nTOS.

## 1. Introduction

Neurogenic thoracic outlet syndrome (nTOS) is a relatively common problem which is frequently not recognized [1]. Some investigators believe that nTOS is overdiagnosed. While they may be right in some circumstances, it is far more likely that nTOS is more commonly underdiagnosed. The diagnosis of nTOS is made by understanding the total clinical picture, and that requires a complete history and physical examination [2]. There is no single test which unequivocally allows the diagnosis to be made in a definitive fashion, but certain imaging modalities may be confirmatory. Symptoms of nTOS are similar to symptoms caused by a wide variety of other conditions. Furthermore, a patient may have multiple coexisting conditions, and the challenge is to identify the etiology of various parts of the symptom complex before making recommendations. These patients look normal in spite of pain and dysfunction. They have often been seen by other physicians, many of whom have decided that the symptoms are psychogenic. The stresses caused by chronic symptoms, coupled with the cursory examinations carried out by some physicians, reinforce the conclusion that the problem is psychogenic. Multiple visits to physicians, who either do not believe nTOS exists or who believe that it is a diagnosis of exclusion, aggravate the emotional problems that these patients experience. Since there are no definitive tests for nTOS, the diagnosis is made by performing complete history and physical examinations to develop an understanding of the total clinical picture. The examining physician needs to understand that TOS is usually neurological and much less commonly vascular.

## 2. Physical Examination in Evaluation of Neurogenic Thoracic Outlet Syndrome (nTOS)

In this day of increasingly complex testing, the patient is often compartmentalized. In the case of nTOS, patients are referred for a determination of the presence or absence of nTOS. It is important to understand that these complicated patients need to have a comprehensive evaluation, including a thorough review of their records, even if there are so many records that they have to be brought on a flatbed truck. This really needs to be done, even though it takes a significant amount of physician time, with no payment for the physician who performs this service. After record review, these patients need a comprehensive history and physical examination with the goal of finding out what is wrong with the patient as a whole. Guided by the examination, tests may be offered and therapy planned. The key is to remember that nTOS occurs in patients with anatomic abnormalities [3] and to look for these on physical examination.

The typical history of patient with lower brachial plexus nTOS consists of discomfort and pain of varying intensity at the base of the neck [4]. The pain radiates down the arm through the ulnar aspect of the forearm into the hand. These patients have paresthesias affecting the ulnar distribution of the hand. Severe headaches are associated with lower brachial plexus nTOS [4]. They generally start at the base of the skull and radiate over the top of the head. The headaches are usually related to arm use rather than the time of day. In some patients, the pain may radiate into the anterior chest simulating cardiac angina. Sleeve like numbness of the arm awakens him or her at night. This is associated with pain in the upper extremity and numbness and pain which radiate down the arm and into the ulnar innervated areas of the hand. In more advanced cases, weakness of the hand and loss of dexterity of the fingers frequently develops. There may be muscle atrophy and impaired use of the arm without paralysis. Arm extension and elevation typically aggravates the symptoms. Activity during the day the results in misery at night, whereas quiet days lead to more comfortable nights. Symptoms typically occur after exercise rather than during exercise. This is the most common presentation of nTOS. As you examine the patient, look for tenderness of the band spot described below, paresthesias, weakness of elbow extension compared to flexion, weakness of the intrinsic muscles of the hand, and numbness in the ulnar distribution.

The typical patient with upper brachial plexus nTOS presents with the same pain in the neck experienced by the lower brachial plexus patient, but also has pain radiating upward to the ear. These patients may have pain affecting the face and temple and hemicranial headaches. They may complain of a stuffy ear with a negative otologic examination. The pain radiates to the upper pectoral area and laterally through the trapezius muscle and down the outer arm. On physical exam, these patients often have a Tinel’s sign radiating into the trapezius and rhomboid muscles. Look for thenar weakness and a Tinel’s sign in the neck.

The physical examination starts when the patient is first introduced to the examiner and concludes as the patient walks out of the examination room. The physician needs to figure out what sort of disease the patient has, as well as what sort of patient has the disease. What is the patient’s overall state of wellbeing? What does he or she look like from across the room? Is the patient’s function limited by nTOS or by another condition? Is stress amplifying the symptoms? Each finding on initial observation often adds focus to further examinations. As much as possible, abnormal findings should be confirmed with another observation or another physical test. Nonphysiological symptoms must be sought. Does he or she have abnormal use of the extremities? Does he or she carry a back pack of large purse? Is that compatible with the symptoms? Are there spontaneous motions of the neck or the extremities observed which do not match the history? Are the symptoms incompatible with the physician’s observations? Or are there signs of non physiologic findings, such as breakaway weakness, or contraction of all muscles of the body except the muscle being tested? 

At the conclusion of the evaluation it may be best to schedule another appointment. That appointment might be to follow up on any tests ordered. More important than the tests is the opportunity to reexamine the patient to see how consistent the symptoms and findings are over time, which may be difficult if the patient is from out of town. Another important consideration is to determine patient expectations. Caution is advised if the patient has seen many doctors and wants an operation. Additionally, be wary of patients with unrealistic expectations who characterize previous doctors as incompetent. Appropriate patient selection for surgical intervention is key to obtaining excellent outcomes in the treatment of nTOS [5,6,7].

One must remember that patients with nTOS usually have an underlying structural anomaly with superimposed trauma, and then look for the anomaly [3]. The trauma may have been severe, but more often the trauma is low intensity occupation induced repetitive activity. Compare one arm to the other looking for swelling, atrophy or another source of assymetry. Look for evidence of vasospasm. Unilateral Raynaud’s phenomenon suggests a local problem such as nerve impingement. Presence of vasospasm in the other hand could be subclinical and indicate a systemic process. This can be evaluated in the vascular laboratory with a cold immersion test.

Physical examination should be systematic and begin with the neck [8]. Check range of motion of the neck, with or without axial loading or pretend axial loading. If the patient complains of pain on neck rotation, position yourself so that he or she has to rotate the neck see what you are doing. See if the symptoms are present even when he or she thinks you are examining something else. See if axial loading causes more symptoms. If it does, then check rotation again with the examiner’s hand on the top of the head but without applying pressure. Try to find the stub of a cervical rib in the posterior aspect of the neck. Palpate the neck musculature for spasm which can cause TOS symptoms by itself. Identify which muscles in particular are in spasm, as that can guide subsequent physical therapy and provides a baseline for follow-up. Check the response to gentle fist percussion of the spine; palpate the spine looking for specific sites of tenderness, such as cervical disks. 

Apply gentle pressure to the anterior scalene looking for pain and possibly pain radiating into the arm or paresthesias radiating to the face and ear. Apply gentle pressure to the band spot, a point in the base of the neck anterior to the trapezius where a type 3 band connects to the first rib [3]. Generalized tenderness does not count. Percuss the anterior scalene placed on the stretch and look for radiation from the site of percussion expecting a Tinel’s sign up along the cheek, into the eye and into the pectoralis area in the case of upper brachial plexus nTOS. In lower brachial plexus nTOS, numbness from palpation of the band spot radiates down the arm and into the last two digits. Palpate the area where the pectoralis minor inserts to the coracoid process looking for palpable spasm of the pectoralis minor and possibly Tinel’s sign radiating into the upper extremity. In the few cases where the pectoralis minor is the culprit, one can often feel the pectoralis minor through the relaxed pectoralis major. In all of these tests, point tenderness rather than generalized tenderness constitutes a positive test. 

Examine the shoulders and arms next [8]. Look for evidence of a winged scapula. Palpate the area of the rhomboid major muscle looking for tenderness and spasm. Check the strength of abduction adduction, flexion, and extension of the shoulder looking for evidence of shoulder impingement syndrome, which can often be identified by pain and weakness of external and internal rotation. Palpate the shoulder joint looking for localized tenderness from tendonitis. Look for Raynaud’s phenomenon, particularly if it is unilateral. Check for medial and lateral epicondylitis. Check the motor strength of flexion and extension of the elbow and of the wrist. In patients with lower brachial plexus nTOS, there is generally weakness of elbow extension compared to flexion on the involved side. Dorsiflexion of the wrist should be examined as well, as that is a monitor for upper brachial plexus nTOS. Check deep tendon reflexes. Look for Tinel’s sign over the ulnar nerve at the elbow and median nerve at the wrist. 

The hands are evaluated next. Examine for the possibility of carpal tunnel syndrome by tapping the median nerve at the wrist, checking for atrophy in the thenar eminence in the case of carpal tunnel syndrome, and in the small muscles of the hand in pronounced forms of nTOS. Check the strength of thumb opposition both to the index finger or the little finger. Check for weakness of the intrinsic muscles of the hand using interphalangeal card test and palpation of the hand with fingers spread or held tightly together. Check grip strength and thumb opposition at the same time. Do a sensory examination of the arm using both light touch and light pinprick. Perform Phelan’s test which reproduces the patient’s symptoms in cases of carpal tunnel syndrome. Phelan’s test is performed with the arms at the patient’s side and the elbows flexed at 90 degrees. The dorsal surfaces of the hands are placed so that the wrists are flexed 90 degrees. This position is held for 60 s looking for reproduction of the patient’s symptoms. 

Physical examination maneuvers aimed at specifically evaluating for nTOS should also be performed and include Adson’s test and Elevated Arm Stress Test (EAST) [9]. Adson first described his test in 1927 [10]. It is performed with the patient seated with the arms resting on the knees. The patient takes a long deep breath and elevates the chin and turns the head to the affected side. An alteration or obliteration of the radial pulse was considered to be pathonemonic for the scalenus anticus syndrome. Other observers add abduction and external rotation of the arm as part of the positioning. Adson’s writings indicate that he was using this test to identify patients who had attachment of the lower anterior scalene muscle to the subclavian artery. It may have had some usefulness in identifying this subset of patients, but it does not apply to the other forms of TOS. In addition, the test is positive as often in normal patients as it is in patients with thoracic outlet syndrome.

The elevated arm exercise test or the elevated arm stress test (EAST) is the most reliable test for the diagnosis of nTOS. It is performed by having the patient put both arms in the 90° abduction external rotation position with the shoulders and elbows in the frontal plane of the chest. The patient is instructed to slowly open and close the hands over the course of 3 min. Normal patients can perform the stress test for 3 min with only mild muscle fatigue and minimal distress. Patients with nTOS, on the other hand, commonly find reproduction of the usual symptoms with an increase in pain in the neck and shoulder. There is aching progressing down the arm, and paresthesias develops in the forearm and the ulnar innervated fingers. Those with arterial compression will develop arm pallor with the arm elevated and reactive hyperemia when it is lowered. Those with venous compression may develop cyanosis and swelling associated with the pain. Many patients with nTOS will be unable to complete this test and will drop the arms after only a brief period of exercise. They recognize symptoms as reproduction of the usual symptoms. Patients who have carpal tunnel syndrome may experience some numbness with this test, but it is from compression of the median nerve and the symptoms will be confined primarily to the first 3 fingers with some radiation up the arm. 

One important distinction must be made when evaluating a patient with nTOS. It is possible that a patient with psychosomatic illness as the cause of his or her symptoms is referred for evaluation for nTOS. Diagnosis of psychosomatic illness needs to be made with the same precision as that of nTOS. Frost in 1972 emphasized that these patients should not be characterized as malingering [11]. They are better characterized as having a psychogenic caricature of somatic disability. Their prognosis is good if they do not get an operation. The key concept in evaluation of these patients is to repeat tests of the same function and look for variations in response and disparity between symptoms and physical findings, particularly over time.

## 3. Physical Therapy in Evaluation of nTOS

An important component of evaluation of patients with potential nTOS is physical therapy (PT). The diagnosis of nTOS can be difficult and often involves multiple examinations and tests to differentiate TOS from other implicating diagnoses [12]. This section describes an effective PT assessment of patients with potential nTOS developed over many years of experience with this patient population. The assessment focuses on a specific evaluation and its contribution to implicating nTOS as causation of symptoms and an important factor for determining surgical candidates. 

The subjective history can provide significant clues as to when symptoms are being produced, as well as the instigating or exacerbating factors [9]. The activity that is linked to symptom production can drive the physical exam based on involved anatomy and the effect on the narrow passageways of nerves and blood vessels. The passageways include the scalene triangles, costoclavicular space and the sub pectoral space. Other important pieces of information can be gathered from prior trauma to the clavicle, shoulder pathology affecting scapulothoracic mechanics or cervical pathology. The time of day when symptoms arise can indicate whether there is a tensioning event or a release event. The tension of a nerve produces ischemia and venous pooling around the nerve and it takes 6 h before the return of normal flow [13]. Upon return of normal flow, paresthesia will be present as axons will begin to fire and may explain the release event [14].

nTOS may be difficult to diagnose as traction of the brachial plexus is intermittent, and there may not be constant sensory or motor deficits. Usually, the first signs to present are non-radicular pain and paresthesia. Visual inspection of static posture is noted with regard to head and shoulder position. Movement provides better clues of dysfunction based on what structural mobility occurs or what myofascial tension impacts movement. As an example, abduction of the scapula needs to take place during active arm elevation for proper kinematics. A dyskinetic scapula might wing secondary to poor muscle recruitment of serratus anterior or abnormal tightness in pectoralis minor that attaches to coracoid process of the scapula. Therefore, PT evaluation needs to be dynamic to reveal mechanical dysfunction that may be producing traction and provoking symptoms. This also sets the PT examination apart from what the function of other disciplines in diagnosis of these patients. 

The PT physical examination involves mobility tests for provocation or functional movement. Testing of the first rib is the most imperative. A spring test in sitting position can reveal both first rib mobility with end feel as well as pain provocation. The Lindgren test is a very reliable test for an elevated first rib and involves cervical rotation and contralateral side bending. An elevated first rib will prevent available movement of C7. The Roos test and the Cyriax maneuver are both provocation tests that use scapular position for indication based on result. Roos test places the patient in an abducted, retracted and depressed scapular position and involves having the patient open and close their hands for 3 min, increasing vascular demand. A test is considered positive if the symptoms are reproduced. The Cyriax maneuver involves unloading the shoulder girdle with the examiner standing behind the patient and bringing the shoulder girdle into elevation. This test is also held for 3 min and considered positive if tingling is reproduced. When normal flow through a nerve is restored, axons will fire producing paresthesia, so the test is looking for a release event based on scapular position. 

Vascular tests include assessment of the radial pulse and placement of the patient in a certain position. Adson’s test, which looks for the disappearance of the radial pulse with a patient’s arm being passively extended while the patent extends and rotates their head toward the examiner. They are asked to hold their breath while assessing for the disappearance of the radial pulse and can implicate the scalenes. Eden’s test involves measuring the radial pulse while the examiner tractions the arm and compresses the clavicle. It can implicate costoclavicular space compression. Wright’s test looks specifically at the sub pectoral passageway and involves hyperabduction of the arm to tension the pectoralis minor and subsequent reproduction of symptoms or change in radial pulse. 

Specific neural tension tests of the ulnar, median and radial nerve can provide information about sensitivity to mechanical loading [15]. Despite evidence that the roots of C5, C6, and C7 are fixed to transverse processes [16] and will not always be as sensitive and specific in diagnosing nTOS, neural irritation rarely allows for mobility of the nerve without a response. Furthermore, neural mobilization tests can try to differentiate between lesions in the proximal and distal parts of the nerve by changing the pressure at tension points and evaluating the patient’s response to the change in tension.

Functional testing involves assessment of the anatomical structures that make up each passageway. Assessment in the cervical spine is also crucial for differentially diagnosing pure cervical pathology versus a residual effect from changing joint mechanics or muscle recruitment. Loading tests such as a Spurling test and unloading tests such as manual traction can provide significant information as to pure cervical related pathology. Assessment of segmental joint mobility can reveal hypo or hypermobility issues at each joint that can then be investigated through their adjacent level relationship or muscle synergistic patterns. Special attention is paid to the cervicothoracic junction as the level where cervical mobility meets thoracic rigidity. Assessment continues down into the thoracic spine measuring both joint position and mobility. Rib articulation with the thoracic spine and spring mobility test can identity dysfunctional structures. Other important joints to assess for mobility are the sternoclavicular and acromioclavicular and their connection to scapular thoracic mobility. Finally, palpation of muscles that form borders of the aforementioned passageways is important to determine length tension as well as provocation, paying special attention to hypertrophy and hypertonia [17]. Comparison with the asymptomatic side can be used for indication of dysfunction. 

Once dysfunctions are identified, an appropriate treatment plan can be constructed to influence the narrow passageways through which the neurovascular tissue has become irritated. Objective improvements of these dysfunctions can be measured and compared with overall improvement of the patient’s symptoms. As a basis, some objective improvement should occur during a 6-week period of consistent non-operative management. Resolution of symptoms that allow for return to function is the long-term goal. However, those patients who do not make any or enough improvement can become candidates for surgery. The surgical procedure of removing the first rib and resecting the anterior scalene is designed to improve the space the brachial plexus has to descend into the arm. So, for the operation to have an optimal outcome, the patient needs to have had direct or indirect dysfunction of the structure that is common to all passageways where compression or traction can occur—the first rib. 

The physical therapist’s role in recognizing candidates for whom an operation will produce the best outcome, is based on his or her clinical examination, as well as the candidate’s response to the physical therapy treatment program. Non-operative management that has taken place over the 6-week period allows for ongoing assessment of anatomy and changes to the objective findings that treatment has been trying to influence. Treatment involves a comprehensive approach of manual therapy directed at bone or joint position, soft tissue mobilization, and therapeutic exercise focusing on recruitment of stabilization based musculature and inhibition of over-utilized muscles. Protocols specific to the underlying pathology of nTOS should be utilized [6,18]. Postural training should be included in non-operative management [19]. Physical therapy may be successful in treating the symptoms of nTOS such that an operation is not required [19,21,22,23]. Continual access to the patient also allows for assessment of compliance to a home exercise program and changes to body mechanics and postural recommendations during function. As treatment progresses, the physical therapist looks for improvement in objective findings to match at least some symptom improvement. When non-operative treatment has been effective with objective measures, but symptoms respond and revert without linear progression, it is appropriate to recommend these patients for surgical consideration.

## 4. The Role of Imaging in Diagnosis of nTOS

Optimal imaging for diagnosis of neurogenic thoracic outlet syndrome (nTOS) remains controversial. Because the underlying pathophysiology is presumed to be bony, muscular or fibrous compression of brachial plexus fibers, imaging tests revolve around modalities that can either identify these structures or assess the function of nerve fibers traversing the thoracic outlet [3]. Conversely, contrast imaging focused on evaluation of the subclavian artery or vein, while useful for evaluation of arterial (aTOS) or venous TOS (vTOS), are typically only suggestive of associated neural compression.

### 4.1. Plain Films

In patients with suspected nTOS, the recommended initial screening test remains plain antero-posterior radiograms of either the cervical spine or chest. Plain films are widely available, low cost, and result in only minimal radiation exposure. A plain film evaluated by a trained interpreter can efficiently screen for a wide variety of potentially relevant bony anomalies, such as incomplete or complete cervical ribs, elongated C7 transverse processes, anomalous first ribs, and anomalous clavicles.

A retrospective review by Weber [22] of preoperative imaging in 400 surgically treated TOS patients revealed that of the 219 with neurogenic TOS, 23% had a cervical rib and 10% had another bony anomaly (first rib, clavicle) [23]. Cervical rib presence was significantly higher in nTOS patients than those with other forms of TOS (23% vs. 16%, *p* < 0.05), while there was no significant difference between the groups with respect to other bony anomalies (10% vs. 7%, *p* = 0.20). In comparison, large population based studies of routine chest films have identified cervical ribs in only 1% of healthy subjects. 

While identification of a bony anomaly, particularly a cervical rib, on plain film may be suggestive of a possible associated nerve compression syndrome, no compelling data exists regarding sensitivity, specificity or accuracy of this finding when compared either to operative findings or to emerging gold standard imaging tests such as CT or MRI. In particular, the absence of a bony anomaly on plain film should not be interpreted as lessening the likelihood of thoracic outlet compression syndrome.

### 4.2. Computed Tomography

**Figure 1:**
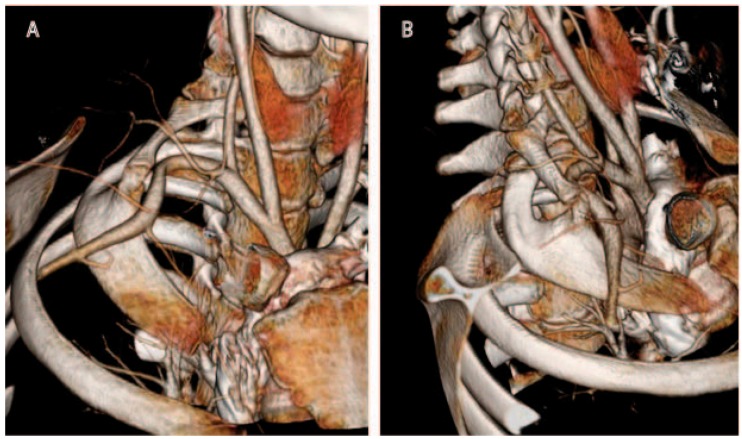
3D reconstruction of computed tomography (CT) arteriography, demonstrating a right-sided cervical rib fused to a broadened first thoracic rib, with anterior displacement of the subclavian artery. (**A**) antero-posterior view; (**B**) oblique view.

Computed tomography (CT) possesses numerous major advantages over plain radiography for evaluation of the thoracic outlet [24,25]. Multi-detector CT technology has improved over the years to permit high-resolution (1 mm cut or less) imaging, and computer processing power now enables rapid 3D reconstruction of images by even non-radiologists (Figure 1). Synchronous administration of timed contrast boluses permits evaluation of the arteries and veins, and has become the gold standard for evaluation of the vascular structures in the thoracic outlet. However, although skilled interpreters may be able to identify brachial plexus structures on CT, nerves are not as well visualized on CT as on MRI. 

A crucial aspect to CT imaging for evaluation of TOS is the use of provocative positioning. Images are first obtained with the arms in neutral position at the sides; subsequently, images are obtained with the arms raised above the head in an attempt to elicit narrowing of the thoracic outlet. Mastumura performed CT arteriography and CT venography in 10 healthy patients, both with and without provocative positioning [24]. Importantly, he found moderate to severe venous compression to be essentially universal with arm elevation, but found that arterial compression in healthy volunteers was either absent or minimal. As such, provocative CT findings of venous compression may reflect normal physiology and should not be considered pathognomonic for nTOS, but findings of arterial compression or arterial aneurysm formation should be taken more seriously.

Remy Jardin et al. performed standard and provocative CT angiography in 79 patients with symptomatic TOS, at least 80% of whom had a neurogenic component, and evaluated changes in the morphologies of the three compartments of the thoracic outlet (interscalene triangle, costoclavicular space and subcoracoid tunnel) [26]. Although he identified a variety of changes in bony and arterial positioning with arm elevation, the most significant finding was a statistically significant reduction in the average maximum distance between the clavicle and first rib (34% reduction in females; 24% reduction in males), contributing to significant compression of the neurovascular bundle. 

### 4.3. Magnetic Resonance Imaging

Magnetic resonance imaging (MRI) is gaining importance in the evaluation of the thoracic outlet. In addition to bone and vessels, MRI permits evaluation of the cervical nerve roots and brachial plexus cords; normal and abnormal muscular structures; and presence of abnormal fibrous bands. Just as with CT, images may be obtained with provocative positioning, although the narrowness of the imaging bore may make arm elevation prohibitive for some body types.

Both Yildizgören et al. and Baumer et al. have reported on the MRI-assisted discovery of fibrous bands in the thoracic outlet causing brachial plexopathies, and Muellner et al. reported on the MRI-assisted discovery of an aberrant subclavius posticus muscle narrowing the costoclavicular space [27,28,29]. None of these abnormalities would have been identifiable on CT images or plain radiogram, demonstrating the significant advantage of MRI over CT for visualization of soft tissue abnormalities in the thoracic outlet.

Evolution of MRI technology has yielded particular benefits for visualization of nerves, a potential major advance in the diagnosis of neurogenic TOS. As strength of closed MRI magnets has increased to 3T, novel sequencing techniques (e.g., Short Tau Inversion Recovery (STIR), Spectral Attenuated Inversion Recovery (SPAIR)), steady state with 3D volumetric acquisition) have permitted vastly improved visualization of nerves, a technology termed MR neurography (MRN). The addition of diffusion tensor imaging (DTI) to standard MRN sequences may even permit visualization of individual nerve fascicles. Such techniques may permit direct imaging of nerve compression or impingement by bony, muscular, or fibrous structures [30]. In classic nTOS, MRN may be able to directly visualize anomalies of the lower brachial plexus cords, such as compression, flattening, or neural and peri-neural inflammation. Such findings may support the contention that anatomic correction of the underlying compression syndrome would be therapeutic; conversely, the absence of any visible lower cord anomalies may be grounds for further diagnostic workup into other potential syndromes.

Beyond its importance in visualization of the thoracic outlet and its components, MRI also permits detailed evaluation of surrounding structures whose dysfunction may mimic the symptoms of nTOS, including evaluation of the cervical spine for assessment of spinal stenosis or cervical disc disease, and evaluation of the shoulder for intrinsic joint or tendon disease.

### 4.4. Positional MRI 

Several investigators have documented the feasibility and added diagnostic utility of MRI with provocative positioning. Nevertheless, positional MRI remains rarely used in clinical practice, and its applicability has not been proven in sizable clinical series.

Demondion et al. first reported positional MRI of the thoracic outlet in a proof-of-concept study using 5 cadavers and 12 healthy volunteers [31]. With a 1.5T magnet, he obtained T1 weighted spin-echo sequences first with the arms alongside the body, then with the arms hyperabducted at 135 degrees. In all scans, the various components of the thoracic outlet were well visualized (interscalene triangle, prescalene space, costoclavicular space and retropectoralis minor space). Sagittal sequences were most useful, as they enabled visualization of the nervous and vascular structures in cross section as they traversed the relevant spaces.

In a larger follow up study, Demondion et al. reported on 35 healthy volunteers and 54 symptomatic patients with TOS who underwent 1.5T MRI imaging with arms in both neutral and hyperabducted positioning [32]. Notably, vascular and nervous compression was visualized only with the arms abducted, reinforcing the importance of provocative positioning. The quality of imaging was sufficient to enable quantitative assessment of bony, muscular, and fibrous components of the thoracic outlet (e.g., minimum costoclavicular distance, subclavius muscle thickness). It was also able to demonstrate specific sites of compression from fibrous bands, underlining the fundamental advantage of a discriminating soft-tissue imaging technique such as MRI over CT.

Smedby et al. performed similar testing on 10 healthy volunteers and 7 patients, but used an open MRI scanner with a 0.5T magnet [33]. The use of an open scanner enabled provocative patient positioning without the associated difficulties of fitting into a narrow bore. Sagittal 3D SPGR sequences were obtained and were of sufficient quality to permit visualization of brachial plexus compression and narrowing of the costoclavicular space

### 4.5. Other Diagnostic Imaging

While MRI and CT have proven to be the most widespread and useful forms of imaging for TOS, application of other imaging modalities, including duplex ultrasound and formal angiography, has been described in the literature. In general, these have proven to be of limited utility for neurogenic TOS.

Demondion et al. attempted to use duplex ultrasound to map the brachial plexus in healthy volunteers and obtained satisfactory visualization in 10 of 12 subjects [34]. Imaging quality was best when using high-frequency linear probes transmitting from 10 to 13 MHz. Of note, the costoclavicular space (a common site of compression in neurogenic TOS) was not directly visualizable due to shadowing artifact, and deeper structures such as the eighth cervical and first thoracic nerve roots were difficult to visualize. He concluded that ultrasound imaging was of potential value for purposes of regional anesthetic administration, but less so for diagnosis of bony compression syndromes. He also found that the quality of imaging was highly dependent on both sonographer technique and patient body habitus.

Simon et al. was able to diagnose a case of neurogenic TOS secondary to a fibrous band extending from an elongated C7 transverse process. Imaging in this case was aided by the fact that compression was not due to an overlying bony structure, but rather, a fibrous band, minimizing shadow artifact [35]. The ultrasound diagnosis was subsequently confirmed with MR neurography and intraoperative findings. Thus, while occasional cases of nTOS may be identifiable with ultrasound, the inability to directly visualize the costoclavicular space remains a major limitation of this technique.

Although resolution of CT and MR imaging has steadily improved, intraluminal defects in arteries and veins remain best visualized on formal angiography (arteriography and venography). These tests permit excellent definition of thrombus, dissection flaps, and other intravascular anomalies. Additionally, provocative positioning with contrast administration may elicit evidence of positional compression syndromes (Scherrer et al.) [36]. However, these tests provide no visualization of neurologic structures and are, therefore, of no value for purely neurogenic TOS. Because of its invasive nature, diagnostic angiography for nTOS is only warranted when clinical suspicion exists for a combined thoracic outlet syndrome with both neurogenic and vascular components, and in situations of diagnostic dilemma unsolvable by CT or MRI imaging. 

### 4.6. Electrodiagnosis

In addition to imaging techniques for visualization of thoracic outlet compression, electrodiagnostic evaluation of the innervation of an affected arm may provide corroborating evidence of brachial plexus compression or damage. It may be of particular use in situations in which imaging has been unable to demonstrate compression, but clinical suspicion for thoracic outlet related neuropathy remains high. 

The typical electrodiagnostic findings in nTOS involve lower brachial plexopathy affecting the lower trunk, C8 and T1 nerve roots [37]. On sensory testing, the most commonly affected nerve is the medial antebrachial cutaneous (MABC) nerve, a cutaneous branch of the medial cord that receives most of its fibers from the T1 nerve root and innervates the skin overlying the distal biceps and medial forearm. On motor testing, the most commonly weakened muscle group is the abductor pollicis brevis, a contributor to thumb abduction and a major muscular component of the thenar eminence. This muscle is innervated by the recurrent branch of the median nerve, which receives many of its fibers from the C8 and T1 nerve roots. Atrophy of this muscle can lead to wasting of the thenar eminence noticeable on physical exam.

Tsao et al. reported on pre-operative electrodiagnostic findings in 32 patients with surgically verified nTOS and confirmed that a T1-focused examination, combining MABC and median nerve testing, was abnormal in 89% of study subjects, while response combinations focused on C8 fibers were less sensitive [38]. This finding of a T1 > C8 axonal loss phenomenon is in contrast to most other kinds of lower brachial plexopathies, which more often affect C8 fibers greater than T1 fibers (e.g., post-median sternotomy). As such, careful performance of electrodiagnostic studies including nerve conduction and needle electrode examination may help discriminate between nTOS and other forms of neurologic injury.

### 4.7. Interventional Imaging

For both diagnostic and therapeutic purposes, some practitioners advocate percutaneous neuromuscular blockade of the anterior scalene, middle scalene, pectoralis minor and subclavius muscles [39]. By inducing transient or long-lasting paralysis of these muscles, the interscalene triangle and costoclavicular space widen. Relief of symptoms with successful blockade may therefore be highly suggestive or confirmatory of nTOS.

Attempts at anesthetizing or chemodenervating these muscles based upon surface landmarks alone may lead to accidental somatic block or sympathetic block in up to 10% of patients. Consequently, numerous imaging modalities have been used to help guide muscle blockade, including ultrasound, fluoroscopy, CT, and needle electromyography [40]. Jordan et al. retrospectively reviewed 245 thoracic outlet chemodenervation procedures using electromyography for confirmation of needle tip localization, 77 of which used adjunctive ultrasound and 168 of which used adjunctive fluoroscopy [41]. Patients were evaluated for procedural success and for complications including dysphagia, dyshponia, pneumothorax, or undesired muscle weakness. Overall complication rates were less than 2%, and median duration of clinical benefit was 4.7 months. Mashayekh et al. reported on 106 patients undergoing 146 scalene injections using CT guidance. In all cases, needle tip localization was satisfactory [42]. No major complications occurred, although 11% reported minor complications such as needle site pain, temporary brachial plexus block, undesired muscle weakness, dysphagia and Horner sign.

It should be noted that reliance on positivity of muscular blockade for diagnosis of nTOS is controversial. Although many cases of nerve compression may be relieved with muscular relaxation, other forms of nerve compression (e.g., compression by a fibrous band) may prove refractory. As such, it would be inappropriate to interpret failure to respond to muscular blockade as ruling out the possibility of nTOS.

## 5. Conclusions

Neurogenic TOS is fundamentally an anatomic compression syndrome. As such, the diagnosis is most readily supported by either radiographic imaging of visibly compressed brachial plexus structures or electrodiagnostic findings consistent with sequelae of intermittent compression. Nevertheless, imaging modalities, like any test, remain susceptible to type I and II errors. Some practitioners express concern that overly strict imaging or diagnostic criteria for nTOS may lead to under-diagnosis and under-treatment. Conversely, others believe that visualized compression in the costoclavicular or retropectoral spaces with provocative positioning may reflect normal physiology, thereby leading to over-diagnosis and over-treatment. Therefore, while advances in imaging technology have provided practitioners with a wealth of new information, the formal diagnosis of neurogenic TOS remains firmly rooted in other aspects of evaluation, notably the clinical history and physical exam. 

Managing and treating TOS can be frustrating and rewarding. A complete history and physical examination are required to make an accurate diagnosis. Expert physical therapy assessment is integral in helping to establish the diagnosis and identify patients who will benefit from operative intervention. Imaging is helpful in ruling out other conditions and confirming the diagnosis. With appropriately selected patients, excellent outcomes are achievable for this sometimes difficult-to-manage condition. Younger patients with shorter duration of symptoms and fewer narcotics used enjoy better results in the long-term follow up of patients surgically treated for nTOS [6,43]. Patients with chronic pain syndromes, smoking, age ≥ 40 years, and opioid use have less favorable outcomes [44]. Patients who are on chronic opioids are challenging to diagnose, and unless physical exam and imaging findings are clear and convincing, operative treatment should probably be avoided. Patients who have had a prolonged history of multiple diagnoses and interventions for upper extremity symptoms may also be poor candidates for surgical treatment. Patients with presumptive nTOS often have antecedent history of accidental injury, and may present within the workers’ compensation or disability system. The associated disability may cloud the accuracy of the diagnosis and compensation status correlates with poor outcomes after surgical intervention [45]. It behooves the surgeon to consider that, while in general well-tolerated, operative treatment of nTOS is associated with several risks including injury to the subclavian artery or vein, brachial plexus, or phrenic nerve. Correct diagnosis and careful patient selection are key to successful treatment of patients with this often misdiagnosed condition.
